# Abstracts from The College of Podiatry Annual Conference 2017: Liverpool, United Kingdom. 16-18 November 2017

**DOI:** 10.1186/s13047-018-0246-5

**Published:** 2018-02-22

## O01 Intermittent claudication – how frequently is it misdiagnosed in the primary care sector?

### Anabelle Mizzi^1^, Kevin Cassar^2^, Catherine Bowen^3^, Cynthia Formosa^1^

#### ^1^Faculty of Health Sciences, Podiatry Department, University of Malta, Msida, Malta; ^2^Faculty of Medicine and Surgery, Mater Dei Hospital, University of Malta, Msida, Malta; ^3^Faculty of Health Sciences, University of Southampton, Southampton, UK

**Background**: The commonest symptom of peripheral arterial disease (PAD) is intermittent claudication (IC). This is associated with an increased risk of myocardial infarction, stroke and cardiovascular mortality. Often patients consult general practitioners (GPs) within primary healthcare sectors. These patients are often referred to the vascular surgeon for specialist assessment and possible revascularisation. Misdiagnosis of IC at the primary visit may lead to inappropriate referral and delayed treatment.

The aim of this study was to determine what proportion of patients referred for intermittent claudication by GPs in Malta for specialist vascular assessment actually have vascular disease.

**Methods**: A cross-sectional observational study was conducted, where all patients referred to a vascular clinic in a local hospital between July 2016 and May 2017, due to IC were invited to participate. Individuals who gave informed consent to participate were assessed for PAD by hemodynamic analysis including Doppler waveforms, ankle-brachial pressure index (ABPI), absolute toe pressures and toe-brachial pressure index (TBPI). A full medical history including medications taken to assess current risk factor control and associated participant demographics were noted.

**Results:** A total of 107 participants were recruited. Fifty-five participants (51.4%) had a confirmed diagnosis of PAD with abnormal Doppler waveforms, sub-optimal ABPI and /or absolute toe pressures and TBPIs. Forty-nine participants (45.8%) had been misdiagnosed, since they presented with triphasic waveforms and normal ankle and toe pressures, indicating no significant vascular impairment. Two participants had possible mixed aetiology of symptoms with marginally abnormal hemodynamics and also spinal stenosis. Ten participants (9.3%) were found to have a systolic blood pressure >160mmHg and were referred to the GP since they were not taking antihypertensive therapy. Eighteen (11.5%) participants were referred back to their GP for risk factor management such as antiplatelet and statins after diagnosis of PAD was confirmed.

**Discussion:** Results demonstrate that the diagnosis of PAD cannot be based solely on clinical symptoms of IC but requires further hemodynamic analysis prior to referral to the vascular surgeon. Differential diagnosis from other conditions which may mimic IC, such as spinal stenosis, is necessary to improve the efficacy of referrals. Inappropriate referrals lead to unnecessary clinical load on a relatively small department resulting in delayed routine new appointments with the vascular surgeon.

Doppler waveform analysis, ABPI and TBPIs are essential tests performed by podiatrists as part of the investigation of symptoms of IC. Use of such haemodynamic testing prior to referral to vascular surgery ensures that patients are only referred where haemodynamic abnormalities are detected as well as identifies those patients with more severe disease, ensuring appropriate prioritisation of cases. Furthermore, immediate referral for appropriate risk factor control in patients with confirmed PAD can be implemented and can lead to a reduction in major cardiovascular events. Additionally, effective referral pathways can also be implemented in those patients who present with symptoms similar to IC due to alternative diagnosis. In patients with IC, haemodynamic analysis at primary care level is recommended in order to ensure appropriate and efficient patient referral and decrease the risk of morbidity.

## O02 Ethical practice - the difference between knowing and doing

### Michael Concannon

#### School of Human and Health Sciences, University of Huddersfield, Huddersfield, UK

**Background:** Moral complexities exist in every day health care practice creating conflicting responsibilities in providing care. Health care ethics (HCE) enable an applied practical linkage of theory and practice to create professional behaviour that focuses on service user benefit.

This presentation summarises a PhD thesis which was an exploration of how physiotherapists and podiatrists embodied health care ethics in their practice. The findings presented locates health care ethics pertinent to physiotherapy and podiatry in the context of contemporary practice in the UK.

**Methods:** how the study was performed and statistical tests used

Interpretative Phenomenological Analysis (IPA) as a hermeneutical approach was utilised in order to explore how HCE informs physiotherapy and podiatry practice. Whilst always involving interpretation, this method has the ability to describe the human experience as it is lived. Using a framework embedded in hermeneutic IPA facilitated an inquiry that promotes the participant’s own reflections of experiential practice (phenomenology) and then interpreting them (hermeneutical) in the relevant and wider context.

Purposively sampled individual interviews were carried out (*n*=21) in an attempt to interpret the participants’ lifeworld of embodied HCE. The preliminary findings were taken to one purposively sampled group interview for discussion which contributed to further interpretation.

**Results:** Five themes emerged from the data. The themes indicated that there is a desire by participants to extol ethical practice, but acknowledged various limitations in the reality of achieving this. The participant’s host organisation has a cultural role in contributing to the individual’s actions and behaviours. The individual’s ethical compass can be blinded by the need for cognitive consonance and the utilitarian ethical approach adopted in health care organisations.

The place of empathy has a key role in HCE for clinical reasoning and decision making which may prevent health care practitioners (HCPs) from passively following performance guidelines and checklists. If empathy and virtue ethics can be taught and utilised by HCPs then guidelines may be considered for individual implementation as an outcome, rather than a prerequisite, of ethical decision making.

**Conclusions:** Ethical decision making may be enhanced by reconsidering the education of character virtues including empathy. Empathy is a basic condition and source of morality. As a central component of phronesis, empathy may enable understanding of a service user’s needs and increase motivation for HCPs to act in a caring way, thus making the service user the bearer of an ethical interaction.

**Trial registration:** This research was given ethical ratification via the University of Huddersfield School Research Ethics Panel. There were further approvals granted from local R&D RECs in NHS Trusts.

## O03 Work participation, mobility and foot symptoms in people with Systemic Lupus Erythematosus: Findings of a UK national survey

### Martin Stevens^1^, Karen Walker-Bone^1^, David Culliford^2^, Begonya Alcacer-Pitarch^3^, Alison Blake^4^, Neil Hopkinson^5^, Lee-Suan Teh^6^, Edward Vital^3^, Christopher Edwards^7^, Anita Williams^8^, Lindsey Cherry^2^

#### ^1^MRC Lifecourse Epidemiology Unit, University of Southampton, Southampton, UK; ^2^Faculty of Health Sciences, University of Southampton, Southampton, UK; ^3^NIHR Leeds Biomedical Research Unit, University of Leeds, Leeds, UK; ^4^Private Practice, Dorset, UK; ^5^Rheumatolgy Department, Royal Bournemouth and Christchurch Hospitals NHS Foundation Trust, Christchurch, UK; ^6^Rheumatolgy Department, Royal Blackburn Teaching Hospital, Blackburn, UK; ^7^Faculty of Medicine, University of Southampton, Southampton, UK; ^8^School of Health Sciences, University of Salford, Salford, UK

**Background:** Employment is a key measure of self-worth, granting independence and social esteem. Non-participation in work is associated with poorer health, higher rates of consultation in primary care and higher rates of indebtedness and mortality[1]. Musculoskeletal (MSK) disorders are one of the two biggest causes of long-term work absenteeism in the developed world [2]. Inflammatory rheumatic disorders in particular have been shown to be associated with high levels of work disability such that 20-70% of people with rheumatoid arthritis have become work disabled within 5-10 years of symptom onset [3]. A recent study by Cherry et al [4] has demonstrated that lower limb and foot problems are highly prevalent among people with SLE. Previous work has also shown that these symptoms are associated with substantial morbidity and functional impairment; 61% of people reported that foot pain adversely affected their lives [5-7]. However, to our knowledge, no research to date has specifically investigated the prevalence of, and relationship between, lower limb or foot related complications, mobility and work non-participation in this patient group.

**Main aim:** The aim of this study was to investigate whether foot and lower limb related symptoms were associated with work participation and mobility in people with Systemic Lupus Erythematosus (SLE).

**Method:** A quantitative, cross-sectional, self-reported survey design was utilised. People with SLE from 6 UK treatment centres and a national register were invited to complete a survey about lower limb and foot health, mobility and work participation. Data collected included work status and the prevalence of foot symptoms including the Manchester Foot Pain Disability index (MFPDI). The focus of the analyses was to explore potential relationships between foot health, mobility, sickness absence and work participation.

**Results:** In total 182 useable surveys were returned; 79 (43%) participants reported themselves as currently employed, 71 (39%) not in work for non-health related reasons, and 32 (18%) as retired/long-term absent from work due to SLE and foot symptoms. Work non-participation due to SLE/foot symptoms was significantly associated with difficulty walking (*p*=0.024), past episodes of foot swelling (*p*=0.041), and past episodes of foot ulceration (p=0.018). There was a significant increase in MFPDI scores amongst those not working (mean 18.13, 95% CI: 14.85 to 21.41) compared to those remaining in employment (mean 10.16, 95% CI: 8.11 to 12.21).

**Conclusions:** 29% of people with SLE eligible to work report themselves as being on long-term sick leave or having retired early because of SLE and lower limb or foot problems. Foot symptoms may contribute importantly to work disability in SLE patients. Further research and clinical time should focus on foot symptoms in SLE patients.

**References**:

1. Black C. Working-for-a-healthier-tomorrow, Dame Carol Black’s Review of the Health of Britain’s Working Age Population 2008. Available online: https://www.gov.uk/government/uploads/system/uploads/attachment_data/file/209782/hwwb-working-for-a-healthier-tomorrow.pdf accessed 03.07.17.

2. Health and Safety Executive. Work-related Musculoskeletal Disorder (WRMSDs) Statistics, Great Britain 2016. http://www.hse.gov.uk/Statistics/causdis/musculoskeletal/msd.pdf2016.

3. Verstappen SM. Rheumatoid arthritis and work: The impact of rheumatoid arthritis on absenteeism and presenteeism. Best Pract Res Clin Rheumatol. 2015; 29: 495-511..

4. Cherry L, Alcacer-Pitarch B, Hopkinson N, et al. The prevalence of self-reported lower limb and foot health problems experienced by participants with systemic lupus erythematosus: Results of a UK national survey. Lupus. 2017; 26: 410-6..

5. Otter SJ, Kumar S, Gow P, et al. Patterns of foot complaints in systemic lupus erythematosus: a cross sectional survey. Journal of foot and ankle research. 2016; 9: 10.

6. Williams AE, Crofts G and Teh LS. 'Focus on feet'--the effects of systemic lupus erythematosus: a narrative review of the literature. Lupus. 2013; 22: 1017-23..

7. Williams AE, Blake A, Cherry L, Alcacer-Pitarch B, Edwards CJ, Hopkinson N, Vital EM and the LS. 2017. Patients experiences of lupus related foot problems: a qualitative investigation. Lupus.

## O04 The temporal progression and natural history of intermittent claudication: a review of current evidence

### Anabelle Mizzi^1^, Kevin Cassar^2^, Catherine Bowen^3^, Cynthia Formosa^1^

#### ^1^Faculty of Health Sciences, Podiatry Department, University of Malta, Msida, Malta; ^2^Faculty of Medicine and Surgery, Mater Dei Hospital, University of Malta, Msida, Malta; ^3^Faculty of Health Sciences, University of Southampton, Southampton, UK

**Background:** Intermittent claudication (IC) is the most common symptom of peripheral arterial disease. Due to the benign prognosis in the majority of patients, treatment is generally aimed at risk factor control. However, approximately 15% of patients with IC deteriorate to critical limb ischaemia (CLI), with increased risk of adverse cardiovascular events, amputation and death. Understanding the natural history and the temporal progression of IC, would help in identifying those with increased likelihood of developing CLI. This information is crucial in the clinical decision whether to offer surgical revascularisation. A structured review of the current knowledge related to the temporal progression of symptomatic PAD and the prognosis of IC was conducted.

**Methods:** The PRISMA checklist for systematic reviews was followed. A literature search for potentially relevant articles was performed up to April 2017, in MEDLINE, Cochrane database of systematic reviews, HyDi and ScienceDirect. HyDi is a search portal provided by the University of Malta that allows users to perform a single search through all the library’s resources. No restrictions on publication date or status were applied. Reference lists of review articles were also searched for relevant literature. Search terms were identified after reading publications related to the subject area. Eligible articles published in English, needed to report the natural history of IC with a minimum review period of 12 months, documenting temporal progression of PAD and identification of prognostic factors. Prospective cohort studies are most suitable to investigate the natural history of events however, other methodologies were also considered. Titles and abstracts were first assessed for relevance and design. Full articles were retrieved if they were eligible. Methodological quality of each study was assessed using the Cochrane collaboration checklist. Data retrieved included trial, details of baseline measurements and IC diagnostic criteria, trial outcomes reporting temporal progression and prognostic factors.

**Results:** Overall, 479 articles were retrieved by electronic and hand search. Of these, 63 articles were considered relevant on the basis of title and/or abstract. However, only 7 full-text articles met the selection criteria (5 Prospective cohort studies, 1 randomised trial and 1 retrospective study), reporting temporal progression and prognostic factors. Only one study reported yearly haemodynamic decline (ABPI decline by 0.014), while the rest reported decline at the end of the trial (up to 12 years) with varied results. This method resulted in most studies reporting data only of survivors, possibly leading to selective survival bias with subsequent underestimation of the extent of the progression of atherosclerosis.

**Conclusions:** This review highlights existing inconsistencies and a paucity of scientific evidence related to the temporal progression of IC. The prognosis and progression of PAD is still not clear in published literature. There is a distinct lack of high quality evidence in the literature to guide appropriate decision making in the context of revascularisation in IC. More evidence is needed to be able to not only identify who will deteriorate to CLI, but also to distinguish those who will deteriorate more rapidly than others and accurately predict the time interval until development of CLI.

## O05 Foot orthoses prescription habits amongst podiatrists: An international survey

### Lara Chapman^1^, Anthony Redmond^2^, Karl Landorf^3^, Keith Rome^4^, Anne-Maree Keenan^5^, Robin Waxman^2^, Begonya Alcacer-Pitarch^2^, Heidi Siddle^2^, Michael Backhouse^2^

#### ^1^Department of Podiatry, Harrogate and District NHS Foundation Trust, Harrogate, UK; ^2^Leeds Institute of Rheumatic and Musculoskeletal Medicine, University of Leeds, Leeds, UK; ^3^Discipline of Podiatry, School of Allied Health, La Trobe University, Melbourne, Australia; ^4^Health and Rehabilitation Research Institute, AUT University, Auckland, New Zealand; ^5^School of Healthcare, University of Leeds, Leeds, UK

**Background:** Foot orthoses (FOs) are frequently used to treat a variety of foot and lower limb conditions, and are recommended in several national guidelines. Functional FOs aim to systematically alter abnormal foot mechanics to alleviate symptoms, and are available in either fully customised or prefabricated forms. Prefabricated FOs can significantly reduce manufacturing and clinical costs, but can have comparable mechanical and clinical effects. Despite this, little is known about which types of orthoses are used and the only published survey dates from 2001. Our study aimed to describe the types of FOs currently in use across the United Kingdom (UK), Australia, and New Zealand, and to determine whether the type of FO prescribed varies between common conditions.

**Methods:** This international cross-sectional, online survey hosted on Bristol Online Surveys explored FO prescribing habits of podiatrists and was distributed through professional bodies in the UK, Australia, and New Zealand. Respondents were asked to report which FOs they prescribed most frequently for patients with 24 common presentations and conditions.

**Results:** Two hundred and sixty-four respondents practising in 19 different countries completed the survey; the majority practised in the UK (124), Australia (79) and New Zealand (32). All podiatrists qualified between 1968 and 2016 and 147 (56%) were female. Podiatrists worked in different healthcare sectors and this varied between countries. For example, 64 (81%) of Australian podiatrists worked solely in the private sector compared to 14 (44%) of New Zealand podiatrists and 44 (36%) of UK podiatrists. Forty-two (34%) of UK podiatrists worked solely in the public sector, compared to 3 (4%) of Australian podiatrists and 2 (6%) New Zealand podiatrists.

UK podiatrists prescribed more prefabricated FOs (mean 5.5 pairs per week) than simple FOs (2.7) and customised FOs (2.9). Podiatrists in New Zealand also prescribed more prefabricated FOs per week on average (7.7) than simple (1.4) and customised FOs (2.8), whilst those in Australia prescribed more customised FOs per week (4.5) than simple FOs (0.8) and prefabricated FOs (1.9). UK respondents were more likely to prescribe prefabricated FOs than any other type of FO for 21 presentations and conditions, although customised FOs were more likely to be prescribed for diabetes with peripheral neuropathy, neurological diseases, and neuromuscular conditions. Podiatrists from New Zealand were more likely to prescribe prefabricated FOs for every presentation and condition except tibialis posterior dysfunction, where customised FOs were more likely. In contrast, customised FOs were more likely to be prescribed for most presentations and conditions by podiatrists practising in Australia, although prefabricated FOs were more likely for Morton’s neuroma, forefoot pain, diabetes without peripheral neuropathy, gout, connective tissue disease and falls prevention.

**Conclusions:** FO prescription habits vary depending on the presentation or condition that orthotic management is targeting, and the podiatrist’s country of practice. Overall, prefabricated FOs were more frequently prescribed in the UK and New Zealand, where more podiatrists undertook public sector work, compared to respondents in Australia, where most worked solely in private practice and customised FOs were more common.

## O06 Sensory Profiles of 60 children who have idiopathic toe walking gait

### Cylie Williams^1,2^ Antoni Caserta^1^

#### ^1^Allied Health Services, Peninsula and Monash Health, Melbourne, Australia; ^2^Department of Physiotherapy, Monash University, Melbourne, Australia

**Background:** Idiopathic toe walking (ITW) is a diagnosis of exclusion. It is diagnosed when children over the age of three walk on their tip toes in the absence of a medical condition known to cause, or be associated with toe walking. Sensory Processing Disorder is identified when children and adults are neurologically inundated with sensory input and unable to process and respond in a way enabling learning and socially acceptable behaviours [1]. People who have this disorder may avoid certain encounters or actively seek out experiences to gain sensory input. There is some evidence that children with ITW process sensory input differently to their peers [2]. This research seeks to explore the sensory profiles of children who have attended podiatry services for ITW.

**Method:** A retrospective file audit was undertaken of children diagnosed with ITW across two podiatry departments between 2011 and 2016. The Sensory profile is 125 questions designed to understand sensory processing behaviours of children between the ages of 3 and 14. The scoring template converts raw scores, indicating if the child has a typical performance, more or less probable or definite difference to a normative population group. Performance scores were described in quadrants, Sensory Seeking, Low Registration, Sensory Avoiding or Sensory Sensitivity.

**Results:** There were 60 files sensory profiles extracted. While more children had presented for ITW, not all podiatrists were trained in administration and scoring of the Sensory Profile. The mean(SD) age of children was 5.4 (1.2) years, and there were 29 males, 20 females. The remaining genders were indeterminable due to extraction method. Four children had typical performances on all four quadrants and six scored a more probable or definite difference on all four quadrants. The majority scored a more probable or definite difference on the Sensory Seeking quadrant (*n*=43, 72%), and Low Registration quadrant (*n*=37, 62%). Fewer scored a more probable or definite difference in the Sensory Avoiding (*n*=19, 32%) and Sensory Sensitivity (*n*=23, 38%). Many children also scored a more probable or definite different on two or more quadrants (*n*=36, 60%).

**Discussion:** Few children displayed a ITW gait and typical sensory processing behaviours. Many children displayed more than one cluster of behaviour patterns, particular relating to sensory seeking behaviours like; touching of people or objects excessively, pursuing movement, taking excessive risks or lacking awareness of risk. This potentially points to some children developing a toe walking gait in response to a need for excessive movement, therefore bouncing from toe walking gait may be a stimulus. The different sensory profiles may impact the success or acceptability of different treatment options. Future research on sensory gating measurement in this population should be considered.

**Conclusion:** ITW is a complex gait pattern that commonly presents to podiatrists. Better understanding the potential reasons children adopt this gait may be the key to better outcomes. Podiatrists should consider screening children with ITW for sensory processing difficulties and refer as needed.

**References**:

1. Dunn, W., Sensory Profile: User's manual. 1999, San Antonio TX: Psychological Corporation. 

2. Williams, C.M., et al., Is idiopathic toe walking really idiopathic? The motor skills and sensory processing abilities associated with idiopathic toe walking gait. J Child Neurol, 2014. 29(1): p. 71-8.

## O07 The UK burden of foot and ankle pain on GPs: An observational overview

### Rachel Ferguson^1^, Daniel Prieto-Alhambra^2^, Andrew Judge^2^, Rafael Pinedo-Villanueva^2^, Antonella Delmestri^2^, Nigel Arden^2^, Catherine Bowen^3^

#### ^1^Podiatry Services, Solent NHS Trust, Portsmouth, UK; ^2^Nuffield Department of Orthopaedics, Rheumatology and Musculoskeletal Diseases, University of Oxford, Oxford, UK; ^3^Faculty of Health Sciences, University of Southampton, Southampton, UK

**Background:** The National Health Service (NHS) is increasingly stretched driving a need for change. Policymakers require estimates of future demand for foot care to instigate policies but there is little data available to produce such estimates. Given that pain is often the driving factor in why, especially in the older population, patients consult their GPs relative to feet and ankles, surprisingly there is a corresponding lack of reports on podiatry. The aim for this investigation was to provide UK wide population level data and improved understanding of the frequency of foot and ankle pain recorded in general practice.

**Methods:** The investigation followed a population-based, retrospective cohort design. The patient population was drawn from the Clinical Practice Research Datalink (CPRD) between January 2010 and December 2013, the most recent 4-year period when the study concept was formulated, in order to provide the most up-to-date information possible. All the CPRD data were collected prospectively. Participating GPs data included medical diagnoses, lifestyle information, drug prescribing, referrals and tests data covering 11.3 million patients at present.

Period prevalence and prevalence for recorded foot and ankle pain were the primary outcome measures. Prevalence for recorded foot and ankle pain was stratified by age, gender and different sub-groups of causes for foot and ankle pain. Further stratification by socio-economic status, lifestyle choices, diabetes and systemic musculoskeletal disease status was performed. An overview of referral patterns and tests requested by GPs was undertaken to identify key patterns. Data were analysed descriptively and presented as frequency, range, mean and standard deviation.

**Results:** For 2010-2013, there was a period prevalence of 7,463 per 100,000 of the population (7.5%) for foot or ankle pain. The total number of recorded episodes of foot or ankle pain over the 4-year period was 567,095 (mean 1.64, range 1 to 135, SD 1.30), giving a prevalence of recorded episodes of foot or ankle pain of 3,738 per 100,000 population (multiple same day recordings are counted once).

The largest groups of patients were female, aged 61-70 years and had a READ code of ‘foot pain’. Those aged 61-70 place the greatest burden on GPs in terms of referrals and tests implemented. The most common specified referrals for foot pain are to orthopaedics and physiotherapy, with podiatry receiving less than half of those.

**Conclusions:** This work demonstrates that foot or ankle pain is reported at all age groups. Those aged 61-70 years place the greatest burden on GPs in terms of recorded foot and ankle pain encounters and onwards referrals to other health professionals and for tests. This overall burden presenting to GPs is substantial, yet the majority of referrals to manage is not to podiatry services.

It is clear that work must be accelerated to prioritize an education campaign for UK GPs to help them understand the whole range of foot health management options, beyond those of diabetic foot that are offered by podiatrists, and what the referral pathways to podiatry are.

## O08 Survey of ultrasound practice amongst podiatrists in the UK

### Heidi Siddle^1^, Aimie Patience^1^, James Coughtrey^2^, Jean Mooney^3^, Martin Fox^4^, Lindsey Cherry^5^

#### ^1^Leeds Institute of Rheumatic and Musculoskeletal Medicine, University of Leeds, Leeds, UK; ^2^Education Department, College of Podiatry, London, UK; ^3^Margaret Thatcher Infirmary Medical Centre, Royal Hospital Chelsea, London, UK; ^4^Manchester Leg Circulation Service, Pennine Acute Hospitals NHS Trust, Manchester, UK; ^5^Faculty of Health Sciences, University of Southampton, Southampton, UK

**Background:** The use of ultrasound in podiatry practice encompasses musculoskeletal ultrasound imaging, vascular hand-held Doppler ultrasound and therapeutic ultrasound. Currently the practice of sonography is not regulated by the HCPC, there is no requirement in the UK to hold a formal qualification, and the College of Podiatry does not define ultrasound training and competencies.

**Aim and objectives:** The primary aim of this study was, for the first time, to establish the current scope of ultrasound practice amongst podiatrists in the UK.

The objectives were to:

- Determine the current use of ultrasound within podiatry practice.
- Determine the current training received by podiatrists.
- Determine the current mentorship received and/or provided by podiatrists using ultrasound

**Methods:** A quantitative study utilising a cross-sectional, on-line, single-event survey was undertaken within the UK. An iterative pilot phase was included to test and refine the questionnaire.

The survey was launched by the College of Podiatry through national professional e-newsletters, Specialist Advisory Groups, the Society professional publication (Podiatry Now) and all Society associated social media sites.

**Results:** Completed surveys were received from 284 podiatrists who routinely used various ultrasound modalities in their practice; 173 (70%) use ultrasound as part of their general practice, 139 (49%) for musculoskeletal problems, 131 (46%) for vascular/high risk assessment, 3 (1%) for podopaediatrics and 39 (14%) to support their surgical practice. Almost a quarter of respondents (*n*=62) worked for more than one organisation; the majority (*n*=202, 71%) were employed by the NHS and/or private sector (*n*=118, 41%).

Nearly all (93%) participants report using a hand-held vascular Doppler in their daily practice to assess patients; 216 (82%) to support decisions regarding treatment options, 102 (39%) to provide diagnostic reports for other health professionals, and 34 (13%) to guide nerve blocks.

Ultrasound imaging was used by 104 (37%) participants in their practice primarily to aid clinical decision making (*n*=81) and guide interventions including steroid injections (*n*=67) and nerve blocks (*n*=39). Most (93%) stated they use ultrasound imaging to treat their own patients, while others scan at the request of other podiatrists (*n*=28) or health professionals (*n*=18). Few participants use ultrasound imaging for research (*n*=7) or educational purposes (*n*=2).

Only 32 (11%) participants (*n*=20 private sector) use therapeutic ultrasound in their clinical practice to treat patients presenting with musculoskeletal complaints, namely tendon pathologies.

Few participants (18%) had completed formal post-graduate ultrasound courses. However, completion of formal CASE (Consortium for the Accreditation of Sonographic Education) accredited courses have increased in popularity over the last five years.

40 (14%) of participants currently receive ultrasound mentorship; the majority from fellow podiatrists (*n*=17) or medical colleagues (*n*=15). Over half (*n*=127) of participants who do not have ultrasound mentorship indicated they would like an ultrasound mentor predominantly for the use of ultrasound imaging. 55 (19%) participants report they currently provide ultrasound mentorship for others.

**Conclusions:** Understanding the scope of ultrasound practice, the training undertaken and the requirements for mentorship will underpin the development of competencies and recommendations defined by the College of Podiatry to support professional development and ensure safe practice.

## O09 Multiple Interventions for Diabetic Foot Ulcer Treatment (MIDFUT) Trial Protocol

### Sarah Brown^1^, Jane Nixon^1^, Frances Game^2^, Sylviya Nikolova^3^, Roberta Longo^3^, Elizabeth McGinnis^4^, Nikki Dewhirst^5^, Paul Chadwick^6^, Richard Leigh^7^, Peter Vowden^8^, Edward Jude^9^, Ian Chetter^10^, Shervanthi Homer-Vanniasinkam^5^, Akila Chandrasekar^11^, Richard Lomas^11^, James Wason^12^, Linda Sharples^13^, David Russell^4^

#### ^1^Clinical Trials Research Unit, University of Leeds, Leeds, UK; ^2^Diabetes and Endocrinology, Derby Teaching Hospitals NHS Foundation Trust, Derby, UK; ^3^Leeds Institute of Health Sciences, University of Leeds, Leeds, UK; ^4^Leeds Teaching Hospitals NHS Trust, Leeds, UK; ^5^Leeds Vascular Institute, Leeds Teaching Hospitals NHS Trust; Leeds, UK; ^6^ Education, College of Podiatry, London, UK; ^7^Podiatry, Royal Free Hospital, London, UK; School of Life Sciences, University of Bradford, West Yorkshire, UK; ^9^Diabetes Centre, Tameside Hospital NHS Foundation Trust, Ashton-under-Lyne, UK; ^10^Academic Vascular Surgical Unit, Hull York Medical School, Hull, UK; ^11^ NHS Blood and Transplant Tissue Services, NHS Blood and Transplant, Liverpool, UK; ^12^MRC Biostatistics Unit, University of Cambridge, Cambridge, UK; ^13^Medical Statistics, London School of Hygiene and Tropical Medicine, London, UK

**Background**

In the UK diabetes affects 4.5 million people of whom approximately 2.5% (112,500) have a diabetic foot ulcer (DFU). In 2014-15 NHS England spent an estimated £972million - £1.13billion, 0.7-0.8% of its budget on DFU treatment, not taking into account additional social and public health sector costs. Despite implementation of multidisciplinary care, healing rates for DFUs at 12 weeks with standard therapies are only 7.7-46%. Delays in healing increase the risks of infection, hospitalisation and amputation. Of those who do not achieve 50% reduction in ulcer size at 4 weeks, an estimated 30-91% will not heal by 12 weeks, which is a marker of the need for early adjuvant therapies. Several treatment options are available for hard-to-heal ulcers with few randomised comparisons evaluating them. Therefore, there is substantial uncertainty surrounding current best management.

**Aims**

The MIDFUT trial aims to assess the clinical- and cost-effectiveness of hydrosurgical debridement alone, or in combination with (i) negative pressure wound therapy or (ii) decellularised human dermal allograft or (iii) both, in the treatment of hard-to-heal diabetic foot ulcers.

**Methods**

MIDFUT is a NIHR HTA-funded, multi-centre, seamless Phase II/III, open-label, parallel group, multi-arm-multi-stage RCT trial of patients with DFU. The main eligibility criteria are adult patients with diabetes and a chronic DFU or surgical debridement wound or open minor amputation, of at least 1cm2 in area, that shows limited response to standard care, defined as having <40% reduction in index ulcer area over a period of at least 4 weeks.

Target recruitment is 660 patients from at least 24 centres over three years.

Phase II will randomise 324 patients into one of four intervention arms or treatment as usual (TAU) in a 1:1:1:1:2 allocation. At 4 weeks post-randomisation the short term outcome of ulcer area reduction by at least 50%, assessed by clinicians masked to treatment group, will be used to select at most two of the four intervention arms to be taken through to Phase III. These intervention arms will have achieved at least 10% absolute improvement in outcome versus TAU. If more than two interventions show a sufficient response then information on the safety profile and costs of treatments will also be considered.

There will be a seamless transition to Phase III of the trial. Phase III will randomise a maximum of 336 patients to (at most) two intervention arms or TAU (1:1:1 allocation). The Phase III primary outcome will be time to healing of the index ulcer, assessed by clinicians who are masked to treatment group. Secondary outcomes are: proportion of DFU healed by 12/20/52 weeks; re-ulceration; infection; revascularisation; SAEs (e.g. amputation, admission to hospital); QoL (DFU-SF and EQ-5D-5L) at 4/12/20/52 weeks; health resource utilisation.

HRA approval has been granted and NIHR portfolio adopted. The trial is currently seeking multi-disciplinary diabetic foot clinics interested in participation.

**References**:

1. Lavery LA, Barnes SA, Keith MS, Seaman JW, Armstrong DG. (2008) Prediction for healing of postoperative diabetic foot wounds based on early wound area progression. Diabetes Care 31(1): 26-29.

2. https://www.diabetes.org.uk/Upload/Shared practice/Diabetic footcare in England, An economic case study (January 2017).pdf.

3. Sheehan P, Jones P, Caselli A, Giurini JM, Veves A. (2003) Percent change in a wound area of a diabetic foot over a 4-week period is a robust predictor of complete healing in a 12-week prospective trial. Diabetes Care 26: 1879-1882

## P01 Is the medial longitudinal arch stiffness of a foot orthosis influenced by commonly used prescription designs?

### Ian Griffiths^1^, Zakiyyah Bibi Auchoybur^2^, Martin McGeough^3^, Julia Shelton^2^

#### ^1^Sports Podiatry Info Ltd, London, UK; ^2^School of Engineering and Materials Science, Queen Mary University of London, London, UK; ^3^Firefly Orthoses Ltd, Sligo, Ireland

**Background:** Foot orthoses have historically been issued in the belief they may positively improve (or correct) skeletal alignment. Despite practitioners worldwide subscribing to this kinematic mechanism to explain how foot orthoses work, the published literature is rather inconsistent in its support for this. However, data does support the contention that foot orthoses may exert their effects via a kinetic mechanism (i.e. the modification of the location, magnitude, vector or temporal patterns of reaction forces at the foot-orthosis interface). As such great interest should be taken in how the design of a foot orthosis may influence its ability to generate reaction forces. One feature of orthosis design which is likely to be key here is the load-deformation characteristics (stiffness) of the shell.

**Methods:** The choice of material prescribed, its thickness and also its geometry will all affect shell stiffness and the subsequent influence of kinetic parameter changes. In this study we took prefabricated orthoses with semi-rigid polypropylene shells, 3mm in thickness, EUR size 42 (KLM Labs, USA) and compared the load-deformation characteristics in the medial longitudinal arch across three different conditions which represented commonly used prescriptions in clinical practice: [1] unmodified shell [2] shell with addition of 6mm heel raise [3] shell with addition of 4 degree varus extrinsic rearfoot post.

**Results:** The devices were fixed to a flat compression plate and vertically loaded from above with an indenter at the highest point of their medial arch at a rate of 10mm/minute using an INSTRON-5691 (UK) mechanical test machine. The results suggest that the addition of a 6mm heel raise and a 4 degree varus extrinsic rearfoot varus post increased the media longitudinal arch stiffness of the devices by 25% and 35% respectively.

**Conclusions:** Whilst this methodology did not perfectly simulate in-vivo loading (and care must also be taken at this time to extrapolate the findings to devices of different materials, thickness and geometries) it may provide some interesting insight into how commonly used prescriptions are altering the ability of an orthosis to introduce kinetic changes. Considering one possible clinical application of this information, it may be reasonable to conclude that if a patient/athlete complains that their devices are too ‘hard’ a first line option could be to remove any extrinsic posting at the rearfoot – this should decrease the stiffness (increase the compliance) of the device and improve tolerance and comfort.

## P02 A proof of concept study for the investigation of the kinetic effect of variable-hardness insoles on external knee adduction moment during walking

### Simeon Yardely, Lucy Gates, Martin Warner, Catherine Bowen

#### Faculty of Health Sciences, University of Southampton, Southampton, UK

**Background:** There is a clinical agenda for the provision of conservative interventions that reduce first peak external knee adduction moment (EKAM1) of individuals with symptomatic medial compartment knee osteoarthritis. The aim of this preliminary study was to investigate the kinetic effects of a novel variable-hardness insole (VHI) on EKAM1, to provide proof of concept for this device that justifies further investigation in future trials.

**Methods:** One healthy participant with no history of lower limb osteoarthritis (female, age 25 years) was recruited to the study. The effect of VHI on EKAM1 and other kinetic variables during gait, when worn in a plain outer-soled plimsoll (sole hardness ~Shore A50), was measured using a 12-camera Vicon motion capture system (Oxford, UK) with a sampling rate of 100Hz. 3 types of VHI with a lateral-to-medial hardness ratio range of 1.6-2.6 (hardness Shore A25-65), and a control insole (hardness Shore A50), were trialled. Visual analogue scores (VAS) were recorded for participant comfort levels in all shod conditions.

**Results:** The VHI with a lateral-to-medial hardness ratio of 2.6 (Insole 3) reduced EKAM1 by 6.30% during walking (0.80±0.03 Nm/kg), as compared with the control condition (0.85±0.05 Nm/kg). Insole 3 produced 48.09% less medial ground reaction force (GRF), a 11.10% medial shift in centre of pressure (COP) and 57.40% less knee adduction angle at EKAM1, as compared with control. Insoles 1 and 2 produced similar kinetic results to control. Insole 3 (94mm) was rated as more comfortable than the control insole (82mm).

**Conclusions:** This study provides proof of concept for VHI, and demonstrates its potential as an attractive and comfortable alternative to existing load modifying devices that reduce EKAM1 during walking. Kinetic effects for Insole 3 are similar to those of variable stiffness shoes (VSS), suggesting that a gait adaptation to VHI reduces EKAM1. Device structure and individual adaptation mechanisms, as well as lateral-to-medial hardness ratio, influence the effect of VHI on EKAM1. Further research examining the kinetic effects of VHI in a full-scale RCT, ultimately in a symptomatic OA population, is warranted.

## P03 Foot orthoses prescription habits among podiatrists in the United Kingdom for people with rheumatoid arthritis

### Lara Chapman^1^, Anthony Redmond^2^, Karl Landorf^3^, Keith Rome^4^, Anne-Maree Keenan^5^, Robin Waxman^2^, Begonya Alcacer-Pitarch^2^, Heidi Siddle^5^, Michael Backhouse^5^

#### ^1^Department of Podiatry, Harrogate and District NHS Foundation Trust, Harrogate, UK; ^2^Leeds Institute of Rheumatic and Musculoskeletal Medicine, University of Leeds, Leeds, UK; ^3^Discipline of Podiatry, School of Allied Health, La Trobe University, Melbourne, Australia; ^4^Health and Rehabilitation Research Institute, AUT University, Auckland, New Zealand; ^5^School of Healthcare, University of Leeds, Leeds, UK

**Background:** Foot orthoses (FOs) are recommended in national guidelines and frequently prescribed for people with both early (< 2 years) and established rheumatoid arthritis (RA). Prefabricated FOs are cheaper and can exhibit comparable effects to customised FOs, but published literature varies in its support for their use in RA. Little is known about what types of FOs clinicians prescribe. This study aimed to describe FO prescription habits of UK podiatrists for people with RA.

**Methods:** One Hundred and Twenty-four UK podiatrists completed an online survey as part of a wider international survey of orthotic prescription habits hosted on Bristol Online Surveys.

**Results:** Seventy-five (61%) podiatrists were female. Forty-two (34%) podiatrists worked solely in the public sector, 44 (36%) worked solely in the private/independent sector, and 38 (31%) worked across both. Eighty-eight (71%) podiatrists treating people with RA prescribed FOs for this condition.

Fifty-seven (46%) podiatrists were most likely to prescribe prefabricated FOs for early RA, whilst 28 (23%) were most likely to prescribe customised FOs, and 20 (16%) were most likely to prescribe simple FOs. For established RA, 61 (49%) podiatrists were most likely to prescribe customised FOs, 23 (19%) were most likely to prescribe prefabricated FOs, and 17 (14%) were most likely to prescribe simple FOs. This pattern was independent of working solely in the public or private sector. Fifty-six respondents indicated their preferred prefabricated FO brands for early RA, and 28 respondents for established RA. Slimflex FOs, (16 podiatrists), were over twice as likely to be prescribed as other brands for early RA. X-Line was the most commonly prescribed brand for established RA, indicated by 7 podiatrists, followed by Slimflex (6) and Vasyli (6).

Forty-seven (53%) podiatrists who prescribed FOs for people with RA indicated that they prescribed customised FOs for people with early RA at least some of the time, rising to 63 (72%) in established RA. Foam-impression boxes were used to capture 3D foot shape for manufacturing custom FOs by 28 (60%) of participants in early RA and 40 (64%) participants for established RA. The most frequently specified shell materials for customised FOs in early RA were semi-flexible (e.g. high density EVA) and semi-rigid (polypropylene), indicated by 17 (36%) respondents in both cases, whilst semi-flexible materials were most frequently specified for established RA, by 28 (44%) of respondents. Semi-flexible rearfoot posting materials (e.g. high density EVA) were most frequently specified for both stages of RA, by 21 (45%) respondents for early RA and 27 (43%) for established RA. The most frequently used top covers provided cushioning (e.g. Poron or polyurethane) for early RA, and cushioning with modification or offloading to the forefoot for established RA.

**Conclusions:** This study describes the most common FO prescriptions for patients with RA within clinical practice in the UK. Podiatrists were more likely to prescribe prefabricated FOs in early RA but custom FOs for people with established disease. The findings help inform decisions when selecting FOs for future studies and enable clinicians to benchmark their practice against national patterns.

## P04 Ultrasound of subtalar joint synovitis in patients with rheumatoid arthritis: results of an OMERACT reliability exercise using consensual definitions

### Heidi Siddle^1^, George Bruyn^2^, Petra Hanova^3^, Félicie Costantino^4^, Annamaria Iagnocco^5^, Andrea Delle Sedie^6^, Marwin Gutierrez^7^, Hilde Hammer^8^, Elizabeth Jernberg^9^, Mihaela Micu^10^, Ingrid Moller^11^, Carlos Pineda^7^, Bethan Richards^12^, Maria Stoenoiu^13^, Takeshi Suzuki^14^, Lene Terslev^15^, Violeta Vlad^16^, Robert Wonink^17^, Maria Antonietta d’Agostino^4^, Richard Wakefield^1^

#### ^1^Leeds Institute of Rheumatic and Musculoskeletal Medicine, University of Leeds, Leeds, UK; ^2^Department of Rheumatology, MC Groep Hospitals, Lelystad, The Netherlands; ^3^Institute of Rheumatology, Charles University, Prague, Czech Republic; ^4^Hôpital Ambroise Paré, Boulogne-Billancourt, France; ^5^Universita degli Studi di Turino, Turin, Italy; ^6^Azienda Ospedaliero Universitaria Pisana, Pisa, Italy; ^7^Instituto Nacional de Rehabilatacion, Mexico City, Mexico; ^8^Diakljohemmet Hospital, Oslo, Norway; ^9^Virginia Mason Medical Center/University of Washington, Seattle, United States; ^10^Rehabilitation Clinical Hospital, Cluj Napoca, Romania; ^11^Instituto Poal de Reumatologia, Barcelona, Spain; ^12^Institute of Rheumatology and Orthopaedics, Royal Prince Alfred Hospital, Sydney, Australia; ^13^Cliniques Universitaires Saint-Luc Institut de Recherche Expérimentale et Clinique (IREC), Brussels, Belgium; ^14^Japanese Red Cross Medical Center, Tokyo, Japan; ^15^Center for Rheumatology and Spine Diseases, Rigshospitalet-Glostrup, Denmark; ^16^Clinical Hospital Sf. Maria, Clinical Hospital Sf Maria, Bucharest, Romania; ^17^Bergman Clinics, Naarden, The Netherlands

**Background:** Subtalar joint (STJ) disease in patients with rheumatoid arthritis (RA) is notoriously difficult to assess clinically [1, 2] and frequently overlooked in favour of assessing the tibiotalar joint. Pathology in the STJ greatly increases between five and ten years of disease duration and regularly precedes changes in the tibiotalar joint [1, 3, 4].

Ultrasound (US) is an accessible, non-invasive, and relatively inexpensive bedside imaging technique, that is repeatable as many times as required at the time of consultation and is characterised by high patient acceptability. Although US has been shown to have a higher sensitivity for detecting inflammation in patients with RA compared to clinical examination, reliability at the STJ has not been confirmed in patients with hindfoot problems [5, 6].

**Objective:** To evaluate the intraobserver and interobserver reliability of the US assessment of STJ synovitis in patients with RA using OMERACT consensual definitions.

**Methods:** Twelve sonographers (10 rheumatologists and two podiatrists) conducted an US reliability exercise on 10 RA patients with rearfoot pain. The anteromedial, posteromedial, and posterolateral STJ was assessed using B-mode and power Doppler (PD) techniques according to an agreed US protocol and using a 4-grade semiquantitative grading score for synovitis (synovial hypertrophy (SH) and power Doppler (PD) signal) and a dichotomous score for the presence of joint effusion (JE). Each patient was randomly assigned to a sonographer where they were assessed in two rounds, one in the morning and another in the afternoon. The STJs of both feet were included in the US assessment. Intraobserver and interobserver reliability were computed by Cohen and Light kappa. Weighted k coefficients with absolute weighting were computed for B-mode and PD signal. Kappa values of 0-0.20 were considered poor, 0.20-0.40 fair, 0.40-0.60 moderate, 0.60-0.80 good, and 0.80-1 excellent.

**Results:** Intraobserver reliability

Mean weighted Cohen’s kappa and 95% confidence intervals [CI] (range) for SH, PD, and JE, was 0.80 (0.62-0.98), 0.61 (0.48-0.73), and 0.52 (0.36-0.67), respectively. Weighted Cohen’s kappa for SH, PD, and JE in the anteromedial, posteromedial and posterolateral STJ was -0.04-0.79, 0.42-0.95, and 0.28-0.77; 0.31-1, -0.05-0.65, and -0.2-0.69; 0.66-1, 0.52-1, and 0.42-0.88, respectively.

Interobserver reliability

Weighted Light kappa (95%CI) for SH was 0.67 (0.58-0.74), 0.46 (0.35-0.59) for PD, and 0.16 (0.08-0.27) for JE. Weighted Light kappa for SH, PD, and JE was 0.63 (0.45-0.82), 0.33 (0.19-0.42) and 0.09 (-0.01-0.19) for the anteromedial; 0.49 (0.27-0.64), 0.35 (0.27-0.4), and 0.04 (-0.06-0.1) for posteromedial, and 0.82 (0.75-0.89), 0.66 (0.56-0.8), and 0.18 (0.04-0.34) for posterolateral STJ, respectively.

**Conclusion:** In this multi-observer study of the STJ in patients with RA, US is a reliable tool for assessing synovitis of the STJ in RA, using a consensus driven US protocol and agreed scoring system. The most reliable site is the posterolateral probe position. A reliable imaging scoring system would be of potential value in clinical practice and research to monitor the effects of drug therapy and mechanical interventions, such as footwear and orthoses, in patients with RA and hindfoot pain over time.

**References**:

1. Otter SJ, Lucas K, Springett K, et al. Comparison of foot pain and foot care among rheumatoid arthritis patients taking and not taking anti-TNF<alpha>: an epidemiological study. Rheumatol Int 2011; 31: 1515-9.

2. Wechalekar MD, Lester S, Hill CL, Lee A, Rischmueller M, Smith MD, Walker JG, Proudman SM. Active foot synovitis in patients with rheumatoid arthritis: Unstable remission status, radiographic progression, and worse functional outcomes in patients with foot synovitis in apparent remission. Arthritis Care Res (Hoboken) 2016; 68:1616-1623.

3. Vidigal E, Jacoby RK, Dixon AS, Ratliff AH, Kirkup J. The foot in chronic rheumatoid arthritis. Ann Rheum Dis. 1975 ; 34: 292-7.

4. Bouysset M, Tebib JG, Weil G, Lejeune E, Bouvier M. Deformation of the adult rheumatoid rearfoot. A radiographic study. Clin Rheumatol 1987; 6 :539-44.

5. Wakefield RJ, Kong KO, Conaghan PG et al. The role of ultrasonography and magnetic resonance imaging in early rheumatoid arthritis. Clin Exp Rheumatol 2003; 21:S42-S49.

6. Wakefield RJ, J E Freeston, P O’Connor, N Reay, A Budgen, E M A Hensor,P S Helliwell, P Emery, J Woodburn. The optimal assessment of the rheumatoid arthritis hindfoot: a comparative study of clinical examination, ultrasound and high field MRI. Ann Rheum Dis 2008; 67: 1678-82.

## P05 The lived experience of the soft heel cast, management of heel pressure ulcerations in the acute setting, and interpretative phenomenological study

### Joanna Woollard

#### Therapy Services, Royal Free Hospital, London, UK

Prevention of pressure ulceration is a key quality indicator throughout both acute and community settings. Good quality evidence and national guidelines on how to prevent heel pressure ulcer is limited (1), An alternative offloading device a soft heel cast has been introduced into Podiatric practise. This qualitative research study aims to seek the patient perspective of the soft heel cast.

**Methods**

Patients who had been provided with the soft heel cast were purposively sampled via a recruitment letter. The aim was to recruit between six and eight participants. All participants opted in and provided their written consent to take part in the study. The data was collected using the exemplary method for an interpretative phenomenological approach (IPA) (2). All interviews were transcribed and the six stages of analysis appropriate to IPA followed meticulously.

**Results**

﻿The analysis identified three inter-related Superordinate themes. It revealed the intimate relationship between the most complex and powerful theme of pain alongside, 'ergonomics of the optimal offloading device' and the 'participants ability to cope'. Five sub themes were identified and discussed; suffering, comfort, size, identity, and ulceration duration.

**Conclusion**

Pain is a complex and subjective phenomenon which cannot be measured directly rather established by the person experiencing it (3). Pain is unique and often difficult to describe, as a consequence it remains poorly understood and inadequately managed. Quality of life should be considered an equal factor alongside the well known triad of 'how to heal a wound'. This research clearly highlights how patients offer suffer in silence.

## P06 Diagnosis, treatment and monitoring of resolution of acute charcot foot neuroarthropathy in diabetes: a systematic review

### Tiffany Chew

#### Foot Care and Limb Design Centre, Tan Tock Seng Hospital, Singapore

**Background:** Early diagnosis and management of acute charcot neuroarthropathy is vital in avoiding the rapid progression towards foot deformities and complications such as higher risk of ulcerations and amputation. Clinical pathways currently available on assessment, diagnosis and management of acute charcot neuroarthropathy are based on consensus and do not accurately present the evidence. The aim of this report is to systematically review the current evidence on the diagnosis, treatment and monitoring for resolution of acute charcot neuroarthropathy. Future clinical pathways will be proposed based on the current evidence reviewed.

**Methods:** A systematic review of both published and unpublished scientific papers was undertaken. MEDLINE, EMBASE, CINAHL, AMED and Cochrane were searched using relevant search terms and keywords. Unpublished papers were searched on ClinicalTrials.gov. Hand searching via reference list was also performed. The search results were then further refined using a systematic approach as described in Preferred Reporting Items for Systematic Reviews and Meta-Analyses (PRISMA). The selected studies were then evaluated for their methodological quality through critical appraisal tools. A quality assessment of the evidence was performed using the Grading of Recommendations, Assessment, Development and Evaluation (GRADE) approach. A narrative synthesis was then conducted.

**Results:** A total of 24 relevant studies were selected for review. 8 studies were on modalities for diagnosis, 10 on treatment modalities and 6 on modalities for monitoring of acute charcot neuroarthropathy resolution. Most of the studies were only of fair quality with 1 systematic review, 4 randomised controlled trials, 5 cohort studies, 3 case-control studies and 11 case-series.

The systematic review revealed a paucity of research with much existing evidence derived from low quality research. Based on the current evidence reviewed, the ideal modality for diagnosing acute charcot neuroarthropathy is MRI. Treatment aims of achieving bone healing, preventing fractures and deformities appear to be from prompt immobilisation using Total Contact Casting. Treatment withdrawal based on MRI findings is the most accurate and safe modality for acute charcot neuroarthropathy. Alternatively, this can be monitored via X-ray if fractures have been established. Based on the findings, a clinical pathway was suggested for the early detection and optimal management of acute charcotneuroarthropathy.

**Conclusion:** There is a clear lack of evidence supporting clinical practice with gaps between evidence and guidelines in the management of acute charcot neuroarthropathy.The challenge in having a good quality research in this area are likely due to the low incidence of this condition, the long healing period and therefore the long-term monitoring of outcomes. The proposed clinical pathway for its management was based on current best available evidence. Future implementation will seek to ensure standardised care for diabetic patients with acute charcot neuroarthropathy, enabling the prevention of foot deformities.

## P07 A randomised comparative evaluation of clinical and home application to investigate the effectiveness of silver nitrate (AgNO3) (95%) for the treatment of verruca pedis

### Michael Concannon; Grace Parfitt

#### School of Human and Health Sciences, University of Huddersfield, Huddersfield, UK

**Background:** In the UK, incidence of viral warts and verrucae in children and adolescents is estimated at between 3.9% and 4.9%, with both genders being equally predisposed to HPV infection (Leiding, 2012). The National Morbidity Survey data (1991-1992) suggests that almost two million people in England and Wales will see their General Practitioner for the treatment of cutaneous warts each year, at a cost of an estimated £40 million per annum (Thomas et al, 2006). 95% Silver Nitrate is frequently used in clinical practice; however, has not been evaluated to determine its’ clinical effectiveness. In addition, self-administered application of silver nitrate is now commercially available, although awareness of its effectiveness in comparison to the clinical application by a health professional is unknown (Bray, 2007). The aim of this research was to evaluate the clinical effectiveness of using 95% concentration silver nitrate for the treatment of verrucae pedis, comparing professional and self-application methods.

**Methods:** A Single-centre, two-armed randomized evaluation was conducted at the University Of Huddersfield podiatry clinic. 113 participants (101 analysed) with verruca pedis were recruited voluntarily to this study. Participants were randomized to either a clinical group, where silver nitrate was applied by a healthcare professional; or a home group, where silver nitrate was self-applied.

The main outcome measures were; post treatment pain, controlling for pre-treatment pain and resolution of the verruca.

An analysis of covariance (ANCOVA) was conducted on the data, using post-treatment VAS pain as the outcome, controlling for pre-treatment pain. An ordinal logistic regression was also conducted on the data, using treatment result as the outcome, with “success” defined to be the reference category.

**Results:** All participants reported a reduction in pain as a result of the intervention.

A significant reduction in pain was reported in all the participants who presented with pain prior to treatment (mean pain reduction of 3.98 points on the VAS (SD 2.27)).

The study showed no significant difference between the home and the clinically applied treatment of verruca pedis, in either of the primary outcome measures.

There was, however, a substantive difference in resolution between groups, with 34.0% full resolution and 26.4 % partial resolution in the clinical treatment group, and 18.8% full resolution and 37.5% partial resolution in the home treatment group.

**Conclusions:** Silver nitrate is a safe, cost-effective treatment option for verruca pedis, with equal success rates when compared between home and clinical applications. It provides an alternative option for practitioners which is easy to apply and has no contraindications for patient suitability.

**Trial registration:** This clinical evaluation was given ethical ratification via the University of Huddersfield School Research Ethics Panel.

## P08 Podiatric problems caused by narrow fitting shoes; an audit of NHS podiatry consultations stratified by gender

### Catherine Bryer, Michael Pearce

#### Podiatry Services, Oxford Health NHS Foundation Trust, Oxford, UK

**Introduction** – There is evidence that a high proportion of the population wear incorrectly fitting shoes, and women are more likely to experience shoe-related foot pain than men [1]. The most common site of pain is the forefoot [2]. An audit was conducted to gather information on the percentage of patients seen at an NHS podiatry community clinic being treated for pathologies caused by narrow-fitting shoes at the forefoot.

**Methods** – Two podiatrists independently kept a tally of the number of patients treated for a pathology where a shoe could be clinically identified as the cause. Data were collected over a four day period, by two different podiatrists, at different times of year.

**Results** – Overall, 10% of all patients presented with midfoot or forefoot pathologies caused by a narrow fitting toe-box. In musculoskeletal podiatry consultations this increased to 15%, and of those all were female. In at risk consultations, 8% presented with a pathology caused by narrow fitting shoes, of these 60% were female (47% of all podiatry patients are female).

**References**:

1. Oke, F., Branthwaite, H. and Chockalingam, N. (2015) ‘Footwear mismatch – do we wear correct-sized shoes?’, Footwear Science. Taylor & Francis, 7(sup1), pp. S76–S77. doi: 10.1080/19424280.2015.1038617

2. Veves, A., Murray, H. J., Young, M. J. and Boulton, A. J. M. (1992) ‘The risk of foot ulceration in diabetic patients with high foot pressure: a prospective study’, Diabetologia. Springer-Verlag, 35(7), pp. 660–663. doi: 10.1007/BF00400259.

## P09 A new system of measuring idiopathic toe walking treatment outcomes

### Kelly Gray^1^, Cylie Williams^2^

#### ^1^Department of Physiotherapy, The Children's Hospital at Westmead, Sydney, Australia; ^2^Allied Health Services, Peninsula and Monash Health, Melbourne, Australia; ^3^Department of Physiotherapy, Monash University, Melbourne, Australia

**Background:** Idiopathic toe walking (ITW) presents as a gait disorder in children over three years of age. It is an exclusionary diagnosis, made when there are no medical conditions known to cause or be associated with that child walking on their tip toes [1]. ITW can present in up to 5% of children and in both genders [2]. While management commonly focuses on maintaining ankle range of motion, no single treatment that has demonstrated full success in heel-toe gait resolution. Furthermore, there are no consistent outcome measures used within ITW treatment studies to allow for direct comparison between treatments. This preliminary research aimed to develop a recording proforma of items that clinician value as measures of treatment success, when working with children who have ITW.

**Method:** An expert panel of health professionals, who routinely treat children with ITW, were recruited for a three round Delphi Panel using online surveys. The first round of questions collected demographic data and asked participants open-ended questions about how they measured parent/child reported treatment success, treatment adherence, gait, pain, ankle range of motion, balance and strength. Subsequent rounds consisted of statements developed from initial round responses which participants were required to indicate consensus or agreement. Questions and statements were included in the final proforma if >70% of participants indicated consensus or agreement with the assessment method.

**Results:** Participants included one Orthopaedic Surgeon, five podiatrists and four physiotherapists with extensive paediatric experience and who routinely treated children with ITW. No clinicians work in the same clinics. All participants completed three rounds. Consensus was achieved in Round One for four statements, and agreement of an additional three measures in subsequent rounds. Parent/child reported final items included a parent/child report of time percentage spent toe walking and quantification of pain, both using an age appropriate visual analogue scale. Final items relating to a clinician observation or measure included a hybrid gait/strength scale, ankle range of motion and treatment adherence using a ‘percentage of the week’ scale.

**Discussion:** This preliminary research identified measures clinicians consider in evaluation of ITW treatment outcomes. Clinicians identified multi-faceted reporting including parent/child recall and physical examination of; ankle range of motion, gait tasks that rely on heel-toe gait normalisation and lower limb strength. It is unknown if parents and children also consider these items as important outcome measures. Future research is underway to determine reliability of the developed hybrid gait/strength measure and to further understand parent and child views of what constitute treatment success.

**Conclusion:** Clinicians believe there is more to ITW treatment success than just a change in ankle range of motion. Future research will help develop a standard suite of clinician friendly measures to better understand the success of the variety of implemented treatments.

**References**:

1. Williams, C.M et al, Gait Posture, 2010. 32(4): p. 508—11.

2. Engstrom, P, Tedroff, K, Pediatrics, 2012. 130(2): p. 279-84.

## P10 A longitudinal evaluation of potential prognostic indicators of plantar fasciitis (PF) treatment outcome: A pilot study

### Kara Simpson^1,2^, Charlotte Dando^1,3^, Lindsey Cherry^1,3^

#### ^1^Faculty of Health Sciences, University of Southampton, Southampton, UK; ^2^Podiatry Department, Dorset Healthcare University NHS Foundation Trust, Dorchester, UK; ^3^Research and Improvement Department, Solent NHS Trust, Southampton, UK

**Background:** People who have plantar fasciitis (PF) often experience a lengthy and unpredictable response to conservative treatment. The rationale for such variable response remains unclear. The aim of this pilot study was to evaluate the feasibility, potential treatment response variance and resource requirement needed to inform a full longitudinal study of prognostic indicators.

**Methods:** Ethical approval was gained. A pilot study using a positivistic, quantitative approach of sixteen participants with clinician confirmed diagnosis of PF was completed. Three separate recruitment strategies were established (A, B and C). Participants demographic, foot biomechanics and plantar fascia tissue characteristics on ultrasound (US) were recorded at baseline. The biomechanics of interest were; Foot posture index (FPI) and ankle dorsiflexion. The US characteristics of interest were; fascial thickness, biconvexity, presence of calcaneal spur and vascularity. Pain and function were recorded using 100mm visual analogue scale (VAS) on palpation of the PF insertion (VAS1), first step in the morning (VAS2) and after a period of activity (VAS3), the PF Pain and Disability Scale (PFPS) and the Foot and Ankle Ability Measure (FAAM). Participants received a standardized treatment protocol of footwear advice, stretching and pre-fabricated foot orthoses in accordance with published guidelines and local policy. Participants were followed-up at 6 weeks (w) and 12w. Retention rates, recruitment strategies and treatment compliance were analyzed to assess the feasibility of the study. Descriptive analysis explored all variables and the outcome measures were examined for a clinically meaningful response. Primary and secondary outcomes were tested using spearman’s rank to assert for correlations to inform a full study.

**Results:** Fourteen (88%) participants completed baseline, 6w and 12w evaluation. Recruitment strategy A failed to recruit any participants, whilst B was most effective, recruiting 69% (11/16), followed by strategy C at 31% (5/16). The average age of participants recruited was 46.31±7.94 and BMI was 30.76± 5.45. The average FPI and ankle dorsiflexion was 4.1 ± 3.5, 9.8 ± 3.1, respectively. Fascial thickness ranged from 4mm – 7.3mm, 56% showed evidence of biconvexity and none showed any vascularity. At 12w 100% (0/14) of patients had clinical improvement in VAS1 and 86% (12/14) on VAS2 and VAS3. Foot function improved in 93% (13/14) of participants and 79% (11/14) of participants had an improvement in reported pain and disability. Correlational analysis revealed that VAS 2 was the only outcome measure to have statically significant moderate/strong positive correlation with all other outcome measures (VAS1 *r* = 0.770, *p* = 0.01; VAS3 *r* = 0.716 *p* = 0.004; PFDS r = 0.535 *p* = 0.049; FAMM *r* = 0.703, p = 0.005).

**Conclusions:** Outcome measures differ strongly in terms of treatment response. VAS 2 is the most appropriate primary outcome measure to demonstrate potential maximal treatment response. Minimal US vascularity was observed and therefore would be a poor characteristic for inclusion. Recruitment via strategy B is the most effective approach for a study of this kind.

## P11 The repeatability and content validity of an expert-derived clinical diagnostic protocol for forefoot neuroma

### Charlotte Dando^1,2^, Jonathan Bailey^1,2^, Lyndon Jones^3^, Lindsey Cherry^1,2^, Catherine Bowen^1^

#### ^1^Faculty of Health Sciences, University of Southampton, Southampton, UK; ^2^Research and Improvement Department, Solent NHS Trust, Southampton, UK; ^3^Total Foot Health Ltd, Salisbury, UK

**Background:** Metatarsalgia is a common term used to describe pain in between the metatarsal bones of the forefoot. Forefoot Neuroma (FFN) is the nerve as it passes between the metatarsal bones and a cause of metatarsalgia. Current methods of diagnosing FFN are varied and may include interpretations of patient reported symptoms, clinical observations or tests. However, similar approaches are used to also diagnose other forefoot pathology such as bursitis, capsulitis or synovitis, with no clear differentiating factors. Currently, there is limited evidence to support a specific clinical diagnostic protocol for FFN. Previous work by the authors has led to the development of an expert-derived clinical diagnostic protocol for FFN. However the repeatability and content validity of this protocol remained unclear.

**Aim:** To determine the repeatability and content validity of a novel expert-derived clinical diagnostic protocol for forefoot neuroma.

**Method:** Ethical approval was obtained (IRAS ref: 14371). A prospective diagnostic study design was implemented over a 10-month period. Participants with forefoot pain and no peripheral neuropathy were recruited from a single UK NHS podiatry musculoskeletal service.

The expert-derived clinical diagnostic protocol was used by the same podiatrist to determine the presence or absence of FFN. A second podiatrist, with PGCert in foot and ankle ultrasonography, conducted a standardised forefoot ultrasound examination as the reference standard to determine the presence or absence of FFN. Both investigators remained blinded to each other’s findings. A sub group of participants (*n*=9) were invited to attend a second appointment to determine intra-rater repeatability. The data was analysed descriptively to define the population with FFN. The intra-rater repeatability and content validity of the diagnostic protocol was evaluated using percentage agreement, Kappa analysis and Content Validity Ratio (CVR) analyses.

**Results:** Thirty participants were recruited to the study (18=female/ 12=male; mean age 58 years, range 37 to 81 years. Of these, 7 participants (6=female/1=male) had confirmed FNN via diagnostic musculoskeletal ultrasound and 8 were identified as having FNN from the clinical assessment protocol. The participant who was diagnosed with FFN using the diagnostic protocol was diagnosed as forefoot bursitis with ultrasound. Relative to ultrasound, the diagnostic protocol had a specificity score of 87.5% and a sensitivity score of 95.6%. The intra-rater repeatability was k0.58; ‘moderate agreement’. Using the CVR formula, the most valid components of the diagnostic protocol were: burning sensations reported by patient (0.87), mulder’s click (0.87) and paraesthesia (0.73) and the least valid were: no swelling (-0.47), clicking reported by patient (-0.47) and diastasis (-0.47).

**Conclusion:** Overall there is evidence to suggest that the diagnostic protocol for FFN is repeatable and valid. Further analysis and refinement of the diagnostic protocol is required to confirm its clinical utility in practical settings.

## P12 An investigation into the susceptibility of podiatry students to facial microbes, with and without cosmetics usage, post clinical exposure

### Lucy Best

#### Podiatry Clinic, New College Durham, Durham, UK

**Background:** The purpose of this investigation is to establish whether or not podiatrists (in this case podiatry students) may be exposed to an increased amount or type of microbes in a general clinical environment, depending on their cosmetics usage. Existing literature describes and explores the microbial contamination of makeup, the increased exposure of podiatrists to certain microbes via nail and skin dust, and the possible adhesive nature of cosmetics. However, makeup is seldom remarked upon in local NHS Trust policies, and never in an infection control capacity.

**Method:** An investigation was undertaken across all current podiatry BSc students, with facial swabs taken from a control group, pre-patient treatment exposure group and post-patient treatment exposure group. Within these two latter groups were individuals wearing and not wearing cosmetics.

**Results:** Microbiological lab analysis identified mixed Staphylococcus species and some Streptococcus species, which was expected, though no dermatophytes from the exposure groups which was not expected. Statistical analysis revealed no link between cosmetics usage and quantity of microbes identified, either between exposure groups or overall between all makeup wearers and all non-makeup wearers.

**Conclusion:** Statistical power was lower than is ideal, and so it is suggested that a larger scale investigation would be appropriate to confirm or challenge these findings.

## P13 How do podiatrists move in clinical practice compared to allied health colleagues?

### Cylie Williams^1,2^, Georde Vuillermin^1,2^, Ross Iles^2^, Kelly-Ann Bowles^3^

#### ^1^Allied Health Services, Peninsula Health, Melbourne, Australia; ^2^Department of Physiotherapy, Monash University, Melbourne, Australia; ^2^Department of Community Emergency Health and Paramedic Practice, Monash University, Melbourne, Australia

**Background:** A recent survey of 948 podiatrists across Australia, United Kingdom and New Zealand found that up to 76% (*n*=719) reported musculoskeletal pain resulting from their working practices [1]. Injuries were reported in all parts of the body and the most significant injuries were at the low back [1]. The frequency of injuries was higher than dentists [2] and nurses [3]. No research has quantified full day lumbar movement patterns in the workplace. This means, there has been no means of identifying “at risk” behaviors with objective measures. The aim of this research was to identify lumbar movement patterns of podiatrists during a standard work day and compare these with other allied health professionals.

**Method:** All allied health professionals within a metropolitan health care network were recruited between June and December, 2016. Participants wore a movement monitoring system (Vi-Move), enabling lumbar movement and muscle activity recording in real time. Participants completed a visual analogue scale (VAS) at the beginning and end of the work period reporting the intensity of low back pain. Means (SD) of movement patterns and pre-post VAS analysis was undertaken. Podiatrists data were extracted and compared to all other allied health professionals. Pain and frequencies of short term and sustained postures were explored with parametric and non-parametric analysis.

**Results:** Participants included 11 Podiatrists and 82 other allied health professionals including Physiotherapy, Occupational Therapists, Speech Pathologists and Dietitians. The mean (SD) monitoring time was 7.7 (0.6) hours. All professions reported a statistically significant difference in pain at the beginning and end of the working day (*p*=0.04). Podiatrists performed more sustained flexions, extensions, left and right lateral flexions per hour and to a greater degree than allied health colleagues, however these differences were not statistically significant (p>0.05). Podiatrists also spent more time standing and sitting, and less time walking, than the other professions, again, the difference was not statistically significant.

**Discussion:** This is the first study using real time postural monitoring of health professionals. This study has identified a trend towards podiatrists holding more frequent and awkward positions. This may be a reason podiatrist’s have reported frequent low back pain in past studies. Those reporting greater low back pain in past studies commonly worked in private settings, but this research was undertaken in hospital and community health services. Future research is needed with podiatrists in private practices and residential aged care settings to determine if the setting plays a role in movement patterns. Additional numbers of podiatry participants are also needed to determine if movement was setting or professional related.

**Conclusion:** Podiatrists report high levels of low back pain. This is potentially attributable to the postures held during treatment. Clinicians need to prioritise their own health to ensure career longevity and should try to move frequently during treatment, and move the patient to them rather than hold awkward positions for longer times.

**References**:

1. Williams, C.M., et al., Exploring musculoskeletal injuries in the podiatry profession: an international cross sectional study. J Foot Ankle Res, 2017. 10: p. 3.

2. Ayers, K.M., et al., Self-reported occupational health of general dental practitioners. Occup Med (Lond), 2009. 59(3): p. 142-8.

3. Davis, K.G. and S.E. Kotowski, Prevalence of Musculoskeletal Disorders for Nurses in Hospitals, Long-Term Care Facilities, and Home Health Care: A Comprehensive Review. Hum Factors, 2015. 57(5): p. 754-92.

## P14 A quantitative study to investigate the relationship between a toe-brachial index in a supine position versus a seated position

### Siu Keung Wan, Lisa Chandler

#### Podiatry Department, The University of Northampton, Northampton, UK

**Background**: Research suggests that toe-brachial index (TBI) can be significantly more reliable than ankle-brachial index (ABI) when arterial rigidity due to medial artery calcification (MAC) is suspected. The need to place patients in a supine position to calculate both indices can be difficult in certain patient groups, including the elderly, those with respiratory impairment or who are wheelchair-bound. Given a prior study had validated the use of a hydrostatic pressure formula to correct ABI measured in a seated to a supine position, a similar investigation into TBI was considered to be worthwhile for application to diabetic patients or where MAC is suspected. The findings, if clinically validated, would offer healthcare professionals an affordable and reliable tool to identify peripheral arterial disease (PAD) when supine postures render a traditional approach to TBI infeasible.

**Aim**: The primary objective of the study was to investigate whether there is an association between the values of TBI measured with participants in a seated and supine position, and to explore the feasibility of predicting TBI from the blood pressure measurements obtained in a seated position.

**Method**: The TBI of 35 healthy volunteers was measured in both seated and supine positions using an automated photoplethysmography (PPG) device (BASIC 3.4, Atys Medical). Data analysis included a Pearson’s correlation test to determine the linear correlation between TBI in each position. Hydrostatic pressure elimination was then applied to infer the supine values of toe blood pressure from a seated position. Regression tests were undertaken to predict supine TBI. Bland-Altman analysis was conducted to evaluate the agreement between predicted TBI and measured TBI.

**Results**: According to the results of Pearson’s correlation test, a positive and strong linear correlation (r = .598, p < .001) existed between the value of TBI measured in the two positions. In terms of predicting supine TBI in regression analysis, the approach of eliminating hydrostatic effect from toe systolic blood pressure (SBP) returned a larger coefficient of determination (R2 = .467) than the other without elimination (R2 = .358). With this approach, supine TBI can be approximated from the inferred supine TBI multiplied by a coefficient of 0.614 plus 0.309.

**Conclusion**: The study results and analyses indicated a positive correlation whereby the greater the seated TBI, the higher the corresponding supine TBI. TBI can be predicted from the seated position with the use of a hydrostatic pressure formula. However, the predicted values should be applied with caution due to the limited sample size involved and potential influence of other parameters; therefore, further investigation is recommended. A supine position should still be recommended for measuring the index where possible given that the predicted TBI from a seated position is not sufficiently agreeable with a supine TBI to be clinically reliable.

## P15 Introducing a podiatry led integrated care pathway for people with Peripheral Arterial Disease: The PodPAD project

### Lisa Farndon, Oliver Binns-Hall, Kayleigh Knight

#### Sheffield Podiatry Services, Sheffield Teaching Hospitals NHS Foundation Trust, Sheffield, UK

**Background**: Peripheral Arterial Disease (PAD) is a long term condition characterised by atherosclerotic obstruction of the lower extremity arteries, and is a marker of patients who are at increased risk of cardiovascular events including myocardial infarction and stroke, even when it is asymptomatic. The incidence in the over 60s is approximately 20% and this increases with age and other factors including smoking, diabetes and existing coronary arterial disease. In the lower limb PAD can lead to intermittent claudication, foot ulceration and critical limb ischaemia; all of which can result in amputation. As clinicians of the lower limb, podiatrists are able to assess patients for signs of PAD offering treatment, advice and follow on referral to secondary care with the vascular surgery team if needed. The clinical evidence base suggests that this intervention (foot care and advice on diet and exercise and smoking) is highly effective at reducing the progression into acute care and can reduce the incidence of amputation by 60%. It is recommended that all commissioners and providers should have a clear pathway for patients suspected of PAD.

**Method**: We investigated the clinical and patient centred outcomes of people with PAD who attended a new podiatry led clinic. All participants received an enhanced vascular assessment carried out by a podiatrist in conjunction with a review of current risk status associated with this disease (hypertension, high lipids, smoking, low activity levels and BMI). The clinic was run in conjunction with a dietician who spoke to each participant about their nutrition and gave advice on healthy eating. The clinic was delivered from a National Centre for Sports and Exercise Medicine and every participant was encouraged to access these facilities as part of their management plan, if they agreed. This involved a referral to the exercise team who were able to devise a tailored, one to one programme for each participant.

**Conclusions**: Primary and secondary outcomes were clinical: presence of claudication pain, distance walked at 3 and 6 months before onset of intermittent claudication, blood pressure and lipid monitoring, weight management, reduced BMI, and the success of any smoking cessation and activity referral. Quality of life and patient satisfaction were also assessed**.** Twenty one patients were recruited and final results will be available at the end of the study. We will use three case studies to illustrate preliminary findings and benefits to participants who have taken part. The case studies illustrate clinical benefits from this enhanced and joint working approach including increased activity levels, success of smoking cessation advice, improvement in quality of life measures and symptoms experienced from PAD.

**References**:

1. BMJ. Best Practice Guidelines - Peripheral Vascular Disease. BMJ Best Practice. 2012.

2. National Institute for Health Clinical Excellence. Lower Limb Peripheral Arterial Disease: diagnosis and management - Clinical Guideline 1472012.

3. All Party Parliamentary Group on Vascular Disease. Tackling Peripheral Arterial Disease More Effectively: Saving Limbs, Saving Lives2013: Available from: http:// appgvascular.org.uk/media/reports/2014-03-tackling peripheral_arterial_disease_more_effectively__saving_limbs__saving_lives.pdf.

## P16 Feet and gait in VitalDanza® – an exploration of embodied experience

### Chris Morriss-Roberts, John Gregory

#### School of Health Sciences, University of Brighton, Brighton, UK

**Background**: Emerging approaches to embodiment theory within psychotherapy could provide useful insight for physical therapies with regard to the psychological component which is inherent within healing and self-care behaviours (both physical and mental) (Koch & Fuchs, 2011, De Jaegher, Di Paolo, & Adolphs, 2016).

**Method**: Interpretative Phenomenological Analysis (IPA) is a research method which can enrich our understanding of human related factors which play a key role in experience, particularly when applied to phenomena which have greater life impact (Smith, Flowers & Larkin, 2009).

The study aimed to investigate the lived experiences of VitalDanza® with focus on the significance of feet and gait. A voluntary sample of 7 participants who had at least one year experience of VitalDanza® were recruited. Ethical approval was gained from a UK based University Ethics and Research Governance Panel. Each participant took part in a semi-structured interview where reflections around feelings, emotions and sensations involved in and in response to VitalDanza® were explored.

**Results**: The IPA approach was used to analyse the interview data and the 2 superordinate themes which were drawn out were: a) perceived feeling of vitality or intense 'aliveness'; and b) an urge for self-expression through physical gestures and affectionate touch. 6 subordinate themes were also drawn out of the data.

The results indicated that participants noted the combination of movement, music and conscious and affectionate interpersonal encounters being physically and psychologically therapeutic and supporting them in their personal development and in adjustment to life.

These results can be interpreted through the theory of human agentic capability described by Bandura (2006) and neuroplasticity described by Kandel (1998). From a podiatric perspective, the data has relevance within the theory of podolinguistics. Data analysis points to the activity and the barefoot state in which it is completed having a bearing on the psychological and social aspects of movement and dance steps, and additionally the uniqueness of the functional nature of the feet in permitting bipedal movement whilst they also have an erogenous potential (Rossi, 1977, Morriss-Roberts 2013).

**Conclusions**: VitalDanza® technique is an emerging movement based technique with therapeutic potential, and can be considered as an outlet for creative expression, improving social connection and supporting physical and mental health (Martello, 2014).

**References**:

Bandura, A. (2006). Toward a Psychology of Human Agency. Perspectives On Psychological Science, 1(2), 164- 180. 10.1111/j.1745-6916.2006.00011.x De Jaegher, H., Di Paolo, E., & Adolphs, R. (2016). What does the interactive brain hypothesis mean for social neuroscience? A dialogue. Philosophical Transactions Of The Royal Society B: Biological Sciences, 371(1693), 20150379. 10.1098/rstb.2015.0379 Kandel, E. (1998). A New Intellectual Framework for Psychiatry. American Journal Of Psychiatry, 155(4), 457- 469. 10.1176/ajp.155.4.457 Koch, S., & Fuchs, T. (2011). Embodied arts therapies.

The Arts In Psychotherapy, 38(4), 276-280. http://dx.doi. org/10.1016/j.aip.2011.08.007

Martello, P. (2014). About Vital Danza. Retrieved 12 January 2017, from http://vitaldanza.com/about/ Morriss-Roberts, C. (2013). Big and Pumped: Embodied Masculinity in Homosocial Sporting Environments (Ph.D). University of East London. Rossi, W. (1977). The sex life of the foot and shoe. London: Routledge & K. Paul.

## P17 Combined Diabetic Foot And Eye Screening - A Feasibility Study

### Jane Lewis^1^, Keri Hutchison^2^

#### ^1^Cardiff School of Health Sciences, Cardiff Metropolitan University, Cardiff, UK; ^2^High Risk and Acute Foot Services, Cwm Taf University Health Board, Cardiff, UK

**Background**: The majority of non-traumatic amputations are most often caused by PAD, followed by diabetes or a combination of both. People with diabetes are 6 times more likely to have an amputation than those without (up to 20/day in the UK) with both high monetary and human costs. The increased risk for cardiovascular morbidity, such as myocardial infarction and stroke, and increased risk for mortality is observed in both symptomatic and asymptomatic patients. A diagnosis of PAD presents the opportunity to initiate secondary prevention by instituting atherosclerosis risk factor modification.This study aimed to explore the feasibility of combining foot and eye screening within one appointment. It also aimed to evidence the prevalence of asymptomatic PAD, uptake of the additional screening and whether the patient experience welcomed a combined service in the future.

**Method**: Potential participants were recruited whilst attending their retinopathy screening appointments 1 day a week over an 8 week period. Those consenting had a lower limb arterial assessment using an automated device measuring ABI and pulse volume negating the need to rest the patient, and plantar light touch sensation checked, during the waiting time required for pupil dilation to occur prior to retinal screening.

**Results**: Three hundred sixty participants were invited to take part. 89% (*n*=321) accepted the PAD screening and met the inclusion criteria. 12% (*n*=38) were found to have neuropathy; 24.6% (*n*=79) to have previously undiagnosed asymptomatic PAD with a 100% positive patient experience.

**Conclusions**: The combined screening was well-received and prevalence of undiagnosed asymptomatic PAD in this study would indicate targeted screening could allow earlier identification and intervention of potentially treatable limb and life threatening disease.

